# CRISPR/Cas12a-Assisted Dual Visualized Detection of SARS-CoV-2 on Frozen Shrimps

**DOI:** 10.3390/bios13010138

**Published:** 2023-01-14

**Authors:** Siwenjie Qian, Yanju Chen, Xiaofu Wang, Tingzhang Wang, Yang Che, Jian Wu, Zhangying Ye, Junfeng Xu

**Affiliations:** 1 College of Biosystems Engineering and Food Science, Zhejiang University, Hangzhou 310058, China; 2 State Key Laboratory for Managing Biotic and Chemical Threats to the Quality and Safety of Agro-Products, Zhejiang Academy of Agricultural Sciences, Hangzhou 310021, China; 3 Key Laboratory of Microbiol Technology and Bioinformatics of Zhejiang Province, Zhejiang Institute of Microbiology, Hangzhou 310012, China; 4 Key Laboratory of on Site Processing Equipment for Agricultural Products, Ministry of Agriculture, Hangzhou 310058, China

**Keywords:** SARS-CoV-2, food safety, dual detection, CRISPR/Cas12a, anti-contamination

## Abstract

Given the possibility that food contaminated with SARS-CoV-2 might become an infection source, there is an urgent need for us to develop a rapid and accurate nucleic acid detection method for SARS-CoV-2 in food to ensure food safety. Here, we propose a sensitive, specific, and reliable molecular detection method for SARS-CoV-2. It has a mechanism to control amplicon contamination. Swabs from spiked frozen shrimps were used as detection samples, which were processed by heating at 95 °C for 30 s. These preprocessed samples served as the templates for subsequent amplification. A colorimetric LAMP reaction was carried out to amplify both the SARS-CoV-2 target and the MS2 phage simultaneously in one tube. MS2 phage was detected by colorimetric LAMP as the internal control, while SARS-CoV-2 was detected with a CRISPR/Cas12a system. The fluorescence results could be visually detected with an ultraviolet lamp. Meanwhile, uracil was incorporated during the LAMP reaction to provide an amplicon contamination proof mechanism. This test could detect as low as 20 copies of SARS-CoV-2 in one reaction. Additionally, the detection could be finished in 45 min. The test only needs a heating block and an ultraviolet lamp, which shows the potential for field detection.

## 1. Introduction

A severe respiratory disease caused by SARS-CoV-2 was reported in December 2019 and soon became a global pandemic, which has made a huge impact on every aspect of our society [1,2]. SARS-CoV-2 is transmitted mainly from person to person via respiratory droplets generated during normal activities such as coughing, sneezing, and talking [3]. However, there is now a hypothesis that SARS-CoV-2 could be transmitted via frozen food or packaging [4]. Transmission might be possible if a healthy person touches the contaminated foods or food packaging with his/her hands, and then immediately touches the mucous membranes of his/her mouth, throat, or eyes [5]. The survival of SARS-CoV-2 on surfaces of cold-chain foods for about 3 weeks supports this possibility [6,7]. Moreover, Jung et al., found that SARS-CoV-2 contamination on food in local retail establishments may be viable to be transported home [8]. In fact, the United States and China have reported that the SARS-CoV-2 infection has been transported among the staff of major pork and beef processing plants, as well as in the cold-chain, under special conditions [9,10]. Han et al., present a variety of evidence on food contamination and foodborne transmission of SARS-CoV-2 [11]. Yekta et al., also support that food maybe a potential vehicle of SARS-CoV-2 [12]. All these facts suggest that contaminated food should be considered a potential means of SARS-CoV-2 transmission. Therefore, to ensure food safety and curb the spread of SARS-CoV-2, a rapid and accurate method for the detection of SARS-CoV-2 in food is in urgent demand.

A reverse transcription-polymerase chain reaction (RT-PCR) method has been widely employed as the ‘gold standard’ test for SARS-CoV-2. It is ideal for standard central laboratories because of its high sensitivity and reliability. However, RT-PCR requires sophisticated sample treatment, a bulky instrument, and skilled personnel operation, all of which limit its on-site application. Loop-mediated isothermal amplification (LAMP) takes places at a constant temperature. Therefore, a simple heating block is enough to perform the amplification reaction [13]. Reverse transcription LAMP (RT-LAMP) could become an alternative to RT-PCR, and it has the potential to facilitate the development of on-site testing. Several detection assays for SARS-CoV-2 employing RT-LAMP have been presented [14,15,16]. However, there is no information or guidance on how to proceed if there is amplicon contamination. Additionally, the amplicon detection is not sequence-specific. Moreover, the feasibility of SARS-CoV-2 detection in food with RT-LAMP assay has not been reported. For a good on-site LAMP test, we assume the following requirements. First, there should be no, or simplified, sample treatment to shorten the operation time. Second, the amplicons should be detected sequence-specifically to avoid false positive results due to nonspecific amplification, etc. Third, there should be a mechanism to control the amplicon contamination. Finally, an internal control needs to be provided to guarantee the reliability of the detection, avoiding possible false negative results due to faulty operations.

With the discovery of its trans-cleavage activity, the CRISPR/Cas system has been employed as a detection tool for nucleic acids [17]. CRISPR provides sequence-specific endpoint amplicon detection, which is important for molecular detection, especially for on-site testing. Of all Cas proteins employed for nucleic acid detection, Cas12a is perhaps the most important one because it can take double-stranded DNA (dsDNA) as its substrate. For Cas12a, it requires a protospacer-adjacent motif (PAM) to fulfill sequence-specific recognition. In 2019, our group found that Cas12a can also take UUUN as its PAM besides the TTTN sequence [18]. In this way, it should be possible to employ a uracil and uracil-DNA glycosylase system to provide a contamination proof mechanism during the amplification step. Meanwhile, the Cas12a system could still be employed for sequence-specific endpoint detection.

Based on all these considerations, we developed a molecular testing method for SARS-CoV-2 in food samples with high sensitivity, specificity, and reliability. In this study, spiked frozen shrimps were used as detection samples. RT-LAMP was employed as the amplification step, not only due to its isothermal amplification but also for the high compatibility of its polymerase with possible inhibitors in the samples. Both SARS-CoV-2 target and MS2 phage, which was included as an internal control, were amplified simultaneously in one reaction. MS2 phage was detected using colorimetry with a pH-sensitive RT-LAMP mixture based on the hydrogen ions released during the amplification. Additionally, The SARS-CoV-2 target was specifically detected by a CRISPR/Cas12a system. Uracil was incorporated during the amplification to provide an amplicon contamination proof mechanism. In this way, an accurate and reliable approach was developed to detect SARS-CoV-2 on spiked frozen shrimp. This method could contribute to effective quarantine and disinfection by tracing contaminated food.

## 2. Materials and Methods

### 2.1. Materials and Chemicals

dNTPs, recombinant RNase inhibitor, and RNase-free water were obtained from TaKara Biomedical Technology Co., Ltd. (Beijing, China). 10× isothermal amplification buffer, MgSO_4_, antarctic thermolabile UDG, Bst 2.0 WarmStart^®^ polymerases, WarmStart^®^ RTx reverse transcriptase, WarmStart^®^ Colorimetric LAMP 2X Master Mix with UDG, dUTPs, dTTPs, dATPs, dCTPs, dGTPs, 1× NEB buffer 2.1, and Cas12a were purchased from New England Biolabs Inc. (Ipswich, MA, USA). Betaine was purchased from Sigma-Aldrich Co. LLC. (St Louis, MO, USA). Whatman Grade 201 qualitative filter papers were obtained from General Electric Biotechnology Co., Ltd. (Hangzhou, China).

LAMP Primers, the single-stranded DNA (ssDNA) probe of CRISPR, and MS2 phage plasmid (GenBank ID NC 001417) were synthesized by Sangon Biotechnology Co., Ltd. (Shanghai, China). crRNA was synthesized by Sunya Biotechnology Co., Ltd. (Hangzhou, China). (The detailed sequence information is listed in Table S1 in the Supporting Information). The pseudovirus of SARS-CoV-2 containing N gene was purchased from Zoonbio Biotechnology Co., Ltd. (Nanjing, China).

A Virus RNA Extraction Kit was obtained from Lnjnbio Biotechnology Co., Ltd. (Shanghai, China). Swabs were obtained from Hannuo Biotechnology Co., Ltd. (Hangzhou, China). The lysis reagent was obtained from Assure Tech Co., Ltd. (Hangzhou, China). The portable ultraviolet lamp was purchased from Tianmai Henghui Light Source Electric Co., Ltd. (Beijing, China).

### 2.2. Sample Collection and Treatment

Information on the preparation of frozen shrimp samples spiked with SARS-CoV-2 pseudovirus can be found in the Supporting Information. Sterile collection swabs pre-moistened with RNase-free water were used to collect the virus by wiping the surface of the shrimps. The swabs were then placed into sterile tubes containing 1 mL of RNase-free water. Additionally, 1000 copies of MS2 phage (GenBank ID NC 001417), used as the internal control, were added to the sterile tubes before the sample treatment procedure (the reaction concentration was set according to reference [15]).

Different sample treatment methods were investigated. In the case of the first method, heating treatment, the samples were incubated at 95 °C for 30 s. For the second treatment method, lysis reagent from Assure Tech Co., Ltd. (Hangzhou, China) was employed to release the nucleic acids from the samples. In this method, the swab was placed in the sterile tubes containing 1 mL of the lysis reagent and incubated for 5 min at room temperature. For the third treatment method, filter paper purification was used, the same as in our previous work [19]. The captured nucleic acids from 100 μL lysis solution were finally eluted in RT-LAMP reaction mixtures. These preprocessed solutions were immediately placed on ice to cool. The commercial virus RNA extraction kit was employed to extract nucleic acids from swab samples of spiked frozen shrimp as a comparison.

### 2.3. RT-LAMP, Uracil-RT-LAMP, and Colorimetric RT-LAMP Assay

RT-LAMP assay was carried out at 65 °C for 40 min. The 25 μL reaction mixtures contained 1× isothermal amplification buffer, 2 mM MgSO_4_, 8 U DNA polymerase, 0.15 U WarmStart^®^ RTx reverse transcriptase, 0.35 mM dNTP, 0.8 M betaine, primer mixture, (1.6 μM FIP, 1.6 μM BIP, 0.2 μM F3, 0.2 μM B3, 0.4 μM LF, and 0.4 μM LB) and 2 μL templates. For UDG-uracil RT-LAMP assay, dUTP instead of dTTP (and in a different concentration) and 1 U antarctic thermolabile UDG enzyme were included. In this case, the reaction was first carried out at 37 °C for 10 min and then at 65 °C for 40 min. For colorimetric RT-LAMP, 1× WarmStart^®^ Colorimetric LAMP Master Mix and UDG were used. Additionally, the duplex colorimetric RT-LAMP contained two sets of primer mixture targeting SARS-CoV-2 and MS2 phage, respectively.

Real-time LAMP reactions were performed in a QuantStudio™ 3 Real-Time PCR system (Thermo Fisher Scientific Inc., Waltham, MA, USA) with 0.2 μM SYTO 9 fluorescent stain. The threshold time (T_t_) reflected the initial time at which a detectable fluorescent signal was generated. For those experiments with endpoint detection, RT-LAMP reactions could be carried out on a heating block with temperature control.

### 2.4. Endpoint-Specific Detection with CRISPR

Detailed information on endpoint amplicon sequence-specific detection with CRISPR can be found elsewhere [17,20]. In brief, 20 μL of CRISPR reaction mixtures contained 1× NEB buffer 2.1, 0.15 μM Cas12a, 0.6 μM crRNA, 2.5 μM ssDNA probe, and 0.4 U recombinant RNase inhibitor. After RT-LAMP reaction, CRISPR/Cas12a mixtures were mixed with amplicons and kept at 37 °C for 10 min. The fluorescence results were visually observed via a homemade portable device designed by our group [18]. To check the reaction kinetics of CRISPR detection, CRISPR reaction could also be performed in a QuantStudio™ 3 Real-Time PCR system with real-time fluorescence detection.

## 3. Results and Discussion

### 3.1. Primer Design and Sample Treatment

**Figure 1:**
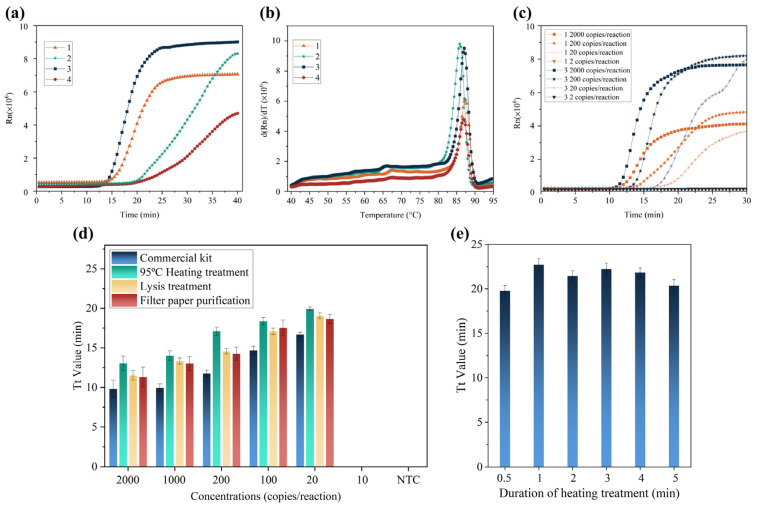
(**a**) RT-LAMP amplification curves of four different primer sets. (**b**) Amplicon melting curves of the primer sets. Numbers 1 and 2 represented the primer set designed by our group; 3 and 4 represent the primer set from the work of Huang et al. (**c**) Comparison of detection sensitivity for the two primer sets with the better amplification performance. The orange curves represent the primer set designed by our group; the dark blue curves represent the primer set from the work of Huang et al. (**d**) Comparison of three sample treatment methods for spiked frozen shrimp samples with different concentrations of SARS-CoV-2. (**e**) Influence of different heating times for RT-LAMP reaction with spiked shrimp samples.

SARS-CoV-2 is an enveloped single-stranded RNA virus with a length of about 30 kb [21]. Since RNA is prone to being attacked by RNase in the environment and degrading from its 5′ terminal, N gene, which encodes nucleocapsid protein at the 3′-end of the virus RNA, might ensure the detection of SARS-CoV-2, even though it is partially degraded [22,23,24]. We investigated LAMP primer sets targeting the SARS-CoV-2 genome (NC 045512.2) in the regions of the N gene. Two primer sets designed by our group and two primer sets from Huang et al.’s work [22] were evaluated. RNA extracted by a commercial kit from a pseudovirus of SARS-CoV-2 was used as a template for RT-LAMP reaction (each reaction contained 100 copies of SARS-CoV-2 RNA). The real-time fluorescence amplification curves and the melting curves of amplicons indicated that all primer sets were capable of amplifying the target genes (Figure 1a,b). One of the primer sets designed by our group and one of the primer sets from Huang et al., had better performance than the other two primer sets. The detection sensitivity of the two better primer sets was further evaluated. Since the two sets of primers had similar amplification efficiency, the primer set designed by our group was employed to perform subsequent RT-LAMP reactions (Figure 1c).

To reduce the time for sample treatment, several simplified methods were investigated, including heating treatment, lysis treatment, and filter paper purification. A series of positive samples was prepared by spiking a known concentration of SARS-CoV-2 pseudovirus on frozen shrimps. As shown in Figure 1d, among these simplified treatment methods, filter paper purification provided a slightly shorter amplification time when the target concentration was the same. However, it needed different steps such as lysis, washing, and eluting procedures. The T_t_ values of RT-LAMP with lysis treatment were similar to those of the filter paper purification. However, lysis treatment required about 5 min to release nucleic acids. The heating treatment took the shortest sample treatment time, and it did not require washing and eluting procedures. We also investigated the effect of different heating times on the amplification efficiency and found that 30 s was almost always the best choice (Figure 1e). Hence, the heating treatment method of 30 s at 95 °C was employed for subsequent SARS-CoV-2 detection.

### 3.2. Optimization of Uracil-Mediated RT-LAMP System

**Figure 2:**
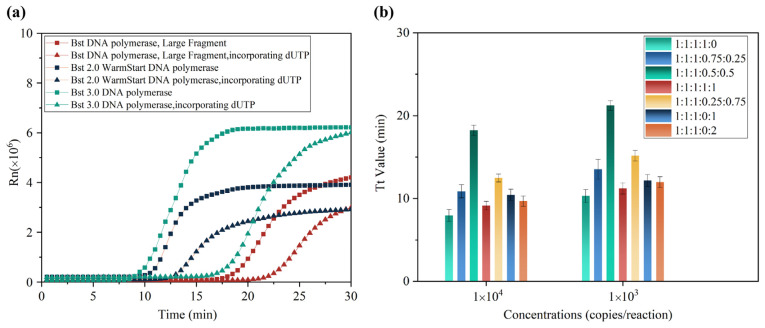
(**a**) Effect of different DNA polymerases and total replacement of thymine with uracil on the amplification efficiency of RT-LAMP reaction. (**b**) Optimization of uracil concentration in RT-LAMP reaction system.

The concentration of LAMP amplicons is higher than that of the PCR reaction. If not handled properly, aerosol-containing amplicons could be spread in the surrounding area and would be difficult to eliminate. Therefore, it is desirable to deploy suitable mechanisms to prevent amplicon contamination. The uracil (U)/uracil-DNA glycosylase (UDG) enzyme system is a well-known approach to prevent amplicon contamination during nucleic acid amplification [25]. However, the total replacement of dTTP with dUTP during the amplification will greatly reduce the RT-LAMP amplification efficiency. To handle this issue, we first investigated the effect of uracil on the amplification speed with different DNA polymerases, including Bst DNA polymerase, Large Fragment, Bst 2.0 WarmStart^®^, and Bst 3.0 DNA polymerase. There were 1000 copies of SARS-CoV-2 RNA in each RT-LAMP reaction. Additionally, the effect of dNTP and total replacement of dTTP with dUTP on amplification speed was checked. The results indicated that Bst 2.0 WarmStart^®^ DNA polymerase provided a better amplification efficiency whether there was dNTP or total replacement of dTTP with dUTP (Figure 2a), while Bst DNA polymerase and Large Fragment had slower amplification speeds, and Bst 3.0 DNA polymerase might produce nonspecific products (results not shown). In this way, Bst 2.0 WarmStart^®^ DNA polymerase was chosen for the next RT-LAMP reaction.

Furthermore, we investigated the effect of a partial replacement of dTTP with dUTP on the amplification efficiency with Bst 2.0 WarmStart^®^ DNA polymerase. Seven combinations with ratios for of dATP, dCTP, dGTP, dTTP, and dUTP of 1 to 1 to 1 to 1 to 0, 1 to 1 to 1 to 0.75 to 0.25, 1 to 1 to 1 to 0.5 to 0.5, 1 to 1 to 1 to 1 to 1, 1 to 1 to 1 to 0.25 to 0.75, 1 to 1 to 1 to 0 to 1, and 1 to 1 to 1 to 0 to 2 were investigated. The final concentration of dATP, dCTP, and dGTP was 0.35 mM. When the concentrations of the five deoxy-ribonucleoside triphosphates were equal, the amplification efficiency was similar to that of an RT-LAMP reaction with dTTP (Figure 2b). Hence, the RT-LAMP reaction system containing 0.35 mM each of the five deoxy-ribonucleoside triphosphates was employed for subsequent testing.

To check the anti-contamination effectiveness of this RT-LAMP reaction system, 1 μL of 1× 10^3^-fold diluted amplicons from previous uracil-mediated RT-LAMP was used as the template for the next RT-LAMP reaction, which contained UDG enzymes. This reaction system was first incubated at 37 °C for 10 min to digest uracil-incorporated amplicons. The UDG enzyme denatured and inactivated at 65 °C, so its effect on subsequent amplification processes can be ignored. We found that no amplification occurred when a 10 min UDG enzyme pretreatment step was included at first. Therefore, the introduction of UDG enzymes and uracil-mediated RT-LAMP could be employed to prevent residual amplicon contamination with our present approach.

### 3.3. Development of UDG-LAMP-CRISPR Detection

Cas12a can take dsDNA as the substrate; therefore, it is suitable for endpoint amplicon detection. On the combination of crRNA and target dsDNA, the trans-cleavage activity of ssDNA will be activated. The ssDNA labeled with fluorescent dye and its quencher on each side can be studied. Due to the fluorescence resonance energy transfer (FRET) effect, there will no fluorescence signal at first. After trans-cleavage, the fluorescent marker and its quenchers will be released. Then, fluorescent signals can be obtained.

**Figure 3:**
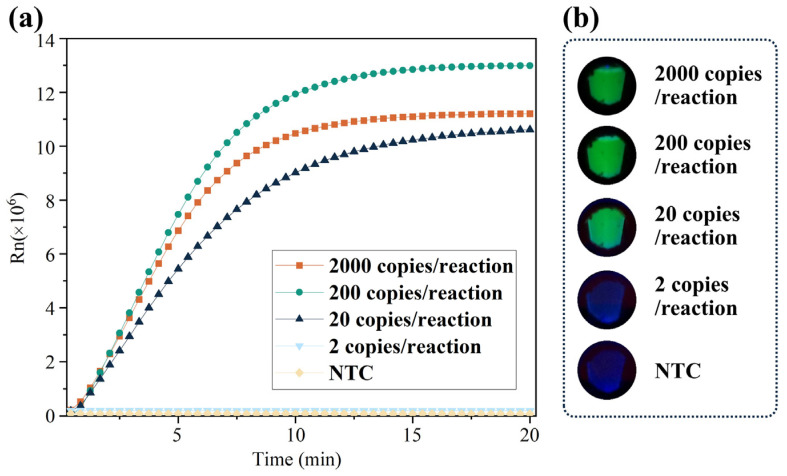
(**a**) Real-time fluorescent signals of the CRISPR reaction. (**b**) Visualized fluorescence results of the CRISPR reaction at 10 min.

The CRISPR/Cas12a reagents were pre-loaded inside the tube lid to avoid uncapping after amplification. Therefore, possible amplicon contamination was avoided. After RT-LAMP reaction, Cas12a reagent was mixed with the amplicons by turning the reaction tube upside down and shaking it several times. We evaluated the detection sensitivity of the UDG-LAMP-CRISPR approach for SARS-CoV-2. A series of known concentrations of samples was prepared. After sampling, the swabs were incubated at 95 °C for 30 s (containing around 1000, 100, 10, and 1 copies/µL of SARS-CoV-2 targets). A total of 2 µL of the mixture was added to the reaction tube to perform reverse transcription and DNA amplification for 30 min, and then the CRISPR reaction occurred over 20 min. As shown in Figure 3a, the CRISPR reaction reached a plateau in 10 min and could detect as low as 20 copies of SARS-CoV-2 target/reaction. The CRISPR reaction could also be performed on a homemade portable device (shown in Figure S1b), through which the results could be observed directly (Figure 3b).

### 3.4. Detection of SARS-CoV-2 with MS2 Phage as Internal Control

Normally, an internal control is employed in molecular diagnosis to avoid false negative results due to operating errors. For qPCR, multiplex amplification could easily be implemented using specific TaqMan probes labeled with different fluorophores. However, although it is possible to sequence-specifically detect LAMP amplicons with a CRISPR/Cas12a system, it cannot perform dual detection in one tube due to non-specific trans-cleavage activity of Cas proteins. Cheng et al., developed a colorimetric system based on CRISPR/Cas12a to analyze telomeres’ repeat DNA and the internal control [26]. Bhatt et al., proposed Cas12-based detection for SARS-CoV-2 and an internal control on lateral flow strips [27]. However, these open detection chambers were prone to amplicon contamination. Misra et al., constructed Cas12a-based detection with a fluorescence readout [28]. Yin et al., developed a lab-on-paper for Cas12a-based diagnostics of SARS-CoV-2 with an internal control [29]. However, these CRISPR reactions were carried out in physically separated chambers. Their work also did not allow for performing multiple detections in one tube. Therefore, we considered exploiting RT-LAMP with a pH-sensitive colorimetric readout and CRISPR-based fluorescence visualized analysis to achieve dual detection in one reaction tube.

**Figure 4:**
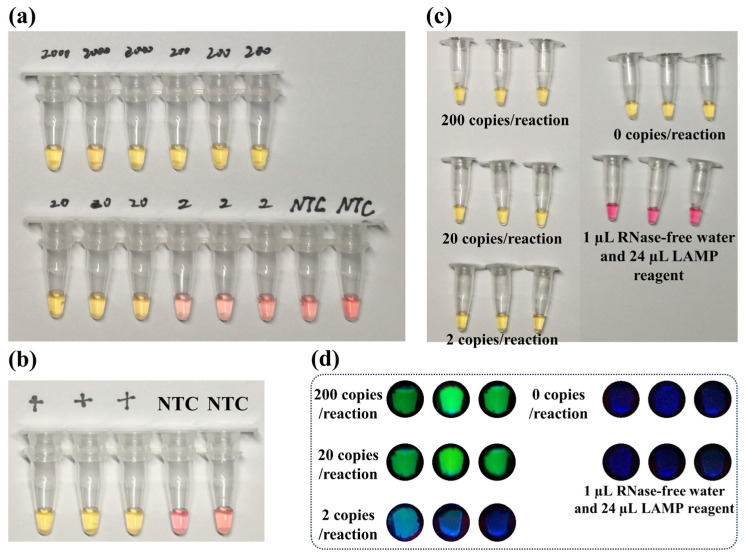
(**a**) Results of colorimetric RT-LAMP for different concentrations of SARS-CoV-2. (**b**) Results of colorimetric RT-LAMP for MS2 phage. (**c**) Results of dual colorimetric RT-LAMP for SARS-CoV-2 and MS2 phage. (**d**) Fluorescence visualized results of CRISPR reaction.

During the extension of the DNA strand, hydrogen ions will be released on the formation of phosphodiester linkage, and, therefore, the pH value of the reaction mixture will be reduced. In 2014, our group developed a method to detect pH changes in real-time during amplification using a micro pH electrode [30]. Although it works well, a pH electrode is required for the detection, which is not convenient for on-site testing. This time, we employed colorimetric RT-LAMP mixtures commercially developed by New England Biolabs Inc. Since the pH of a colorimetric RT-LAMP system is easily affected by sample conditions, the swab was placed in RNase-free water (pH 7.0) to minimize the impact of pH indication in RT-LAMP reactions. We firstly evaluated the detection sensitivity of the colorimetric RT-LAMP reaction for SARS-CoV-2 RNA, which was used as the target. The results showed that as low as 20 copies/reaction could be detected, which was similar to fluorescence-based RT-LAMP. The negative samples remained pink (Figure 4a). The detection feasibility of a colorimetric RT-LAMP reaction for MS2 phage (the internal control) was then evaluated. Fluorescence RT-LAMP served as a contrast. The results showed that colorimetric RT-LAMP could effectively amplify MS2 phage targets (Figure 4b and Figure S2).

Since single-plex detection with the colorimetric RT-LAMP approach was practicable, the dual detection of SARS-CoV-2 and MS2 phage was further investigated. A total of 1 µL of swab samples from spiked shrimp with different concentrations of SARS-CoV-2 (200, 20, 2, and 0 copies) and MS2 phage (1000 copies) was used as the template for a duplex RT-LAMP reaction after heating treatment. The reaction was performed on a homemade portable device, which could also be employed to observe fluorescent signals of CRISPR reactions visually (Figure S1a). The reaction tube containing the reagent mixture was inserted into the portable device to carry out the RT-LAMP reaction for 30 min. Afterward, tubes were taken out to check their color. The tubes containing swab samples and MS2 phage had a color change from pink to yellow, while the tube with water was still pink, which indicated no amplification (Figure 4c). Subsequently, the tubes were put back into the device to carry out 10 min of CRISPR reaction. After the CRISPR reaction, the fluorescent signal in the tube was checked by the naked eye through a 520 nm filter. There was a green, fluorescent signal in a SARS-CoV-2-positive sample, while no fluorescent signal was present in a negative sample. Both tubes with 200 and 20 copies of SARS-CoV-2 showed bright fluorescent signals (Figure 4d). This indicated that the detection sensitivity with the internal control was 20 copies of SARS-CoV-2 per reaction.

**Figure 5:**
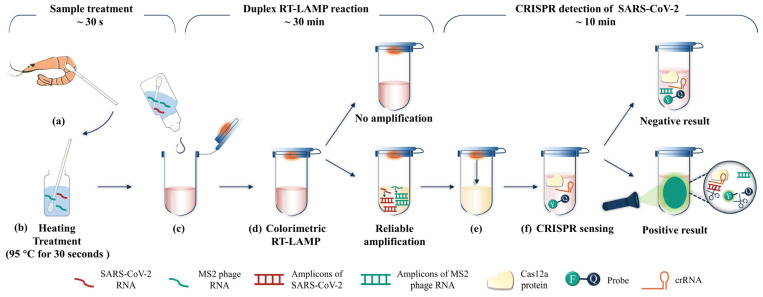
A schematic of the sensitive and reliable detection method for SARS-CoV-2 on spiked shrimp samples. (**a**) Sampling from the surface of shrimp. (**b**) Heating treatment for swab sample. (**c**) Adding one drop of target sample as amplification template. (**d**) Carrying out LAMP reaction at 65 °C. An additional heating step at 37 °C for 10 min will be included before amplification if there is amplicon contamination. (**e**) Mixing the CRISPR reagent which pre-added inside the tube lid with LAMP amplicons. (**f**) Carrying out CRISPR reaction at 37 °C for 10 min and observing the fluorescence results with an ultraviolet lamp.

In this way, a sensitive and reliable testing approach was developed for SARS-CoV-2 detection on spiked frozen shrimps (Figure 5). A sterile collection swab pre-moistened with RNase-free water is used to collect the targets by wiping the surface of the shrimp samples. (Figure 5a). Then, the swab is put in the sterile tube, which contains about 1 mL of RNase-free water, and the mixture is heated to 95 °C for 30 s to release the nucleic acids (Figure 5b). Afterward, one drop of that solution is added to the reaction tube containing RT-LAMP reagent (Figure 5c). The amplification is carried out on a heating block at around 65 °C (Figure 5d). After 30 min, the reaction tube is taken out to check the color of the reaction mixture. A pink color indicates that something is wrong with this test. Should this occur, a new test should be carried out again to ensure a reliable result. A mixture with yellow or orange color indicates that the test has passed the internal control. In this case, turn the reaction tube upside down several times to mix the LAMP reaction mixture with the CRISPR detection reagent, which has been pre-loaded inside the tube lid (Figure 5e). Put the reaction tube on a heating block at around 37 °C (Figure 5f). Finally, check the fluorescence result with an ultraviolet lamp. Bright green fluorescence means a positive result, while no fluorescence represents a negative result. The whole test can be finished in 45 min. Uracil was included during the amplification to provide an amplicon contamination proof mechanism. In case there is amplicon contamination, an additional heating treatment step at 37 °C for 10 min can be included before the amplification step.

## 4. Conclusions

A sensitive and reliable molecular testing approach for SARS-CoV-2 on spiked frozen shrimp was developed. To simplify the operating procedure, swab samples were only treated at 95 °C for 30 s. A duplex colorimetric RT-LAMP reaction was employed to amplify both the SARS-CoV-2 target and MS2 phage simultaneously. MS2 phage, included as an internal control, was detected by colorimetric RT-LAMP, while SARS-CoV-2 RNA was detected with the CRISPR/Cas12a system. Due to its trans-cleavage activity of ssDNA reporters, the CRISPR system could provide sequence-specific detection. The detection results could be visually observed under the illumination of an ultraviolet lamp. According to our experimental results, as low as 20 copies/reaction of SARS-CoV-2 target can be detected. The whole testing was finished in 45 min when there was no residual amplicon contamination. Uracil was incorporated during the amplification to provide an amplicon contamination proof mechanism. Besides disposable components such as swabs, reaction tubes, and reagents, only a heating block with temperature control and an ultraviolet lamp were required for this testing, which makes it a potential test that can be implemented in the field such as at customs, in warehouses, and at supermarkets.

## Data Availability

Not applicable.

## Supplementary Materials

The following supporting information can be downloaded at: https://www.mdpi.com/article/10.3390/bios13010138/s1, Table S1: The sequence information of primers, crRNA and ssDNA probe; Table S2: The sequence information of four primer sets for LAMP; Figure S1: The portable device for LAMP reaction (a), and visualized fluorescence observation (b); Figure S2: The real-time amplification curves of RT-LAMP for MS2 phage RNA (containing 1000 copies/reaction). Figure S3: The real-time fluorescence amplification curves of LAMP reaction after UDG treatment and without UDG treatment. Refs [31,32] are cited in Supplementary Materials.
