# Supplementary material for: CRISPR/Cas12a-Assisted Dual Visualized Detection of SARS-CoV-2 on Frozen Shrimps
Source: Biosensors (Basel). 2023 Jan 14;13(1):138. doi: 10.3390/bios13010138 (PMC9855800; doi:10.3390/bios13010138)
Supplement: Supplementary file 1 [file biosensors-13-00138-s001.zip › biosensors-2048074-supplementary.pdf]

biosensors

Supporting Information

CRISPR/Cas12a-assisted dual visualized detection of SARS-CoV-2 on frozen shrimps

Siwenjie Qian [1], Yanju Chen [1], Xiaofu Wang [2], Tingzhang Wang [3], Yang Che [3], Jian Wu [1,4], Zhangying Ye [1] and Junfeng Xu [2,*]

1 College of Biosystems Engineering and Food Science, Zhejiang University, Hangzhou, 310058, China
2 State Key Laboratory for Managing Biotic and Chemical Threats to the Quality and Safety of Agro-products, Zhejiang Academy of Agricultural Sciences, Hangzhou 310021, China
3 Key Laboratory of Microbiol Technology and Bioinformatics of Zhejiang Province, Zhejiang Institute of Microbiology, Hangzhou, 310012, China
4 Key Laboratory of on Site Processing Equipment for Agricultural Products, Ministry of Agriculture, Hangzhou, 310058, China
* Correspondence: xujunfeng@zaas.ac.cn

Preparation of spiked frozen shrimp

The preparation process of spiked frozen shrimp samples was similar to that reported by our group previously [1]. Fresh shrimp samples were purchased from local supermarket. They were immersed in 75% ethanol for 2 min to eliminate background microorganisms. After washing in RNase-free water, shrimp samples were placed in a biological safety hood under ultraviolet light for 30 min. And then the sterile shrimp were placed in a freezer at -20 °C for 24 hours. A series of gradient concentrations of SARS-CoV-2 pseudovirus suspensions were prepared with PBS. Shrimp samples were incubated in SARS-CoV-2 suspensions for 30 min at 2 °C. Then they were transferred to clean plates and kept for another 30 min to allow viruses attachment at -20 °C.

Analysis of melting curve

For the melting curve analysis, after PCR and LAMP amplification, the reaction mixtures containing amplicons were firstly heated at 95 ºC for 3 min. Then, the temperature of reaction mixtures was dropped to 60 ºC. Melting process was performed at an ascent rate of 0.15 ºC/s from 60 ºC to 95 ºC and the fluorescent signal was recorded every 2 s. The whole operation process was conducted in a QuantStudio™ 3 Real-Time PCR System (Thermo Fisher Scientific Inc., Waltham, MA, USA).

Table S1. The sequence information of primers, crRNA and ssDNA probe.

Category	Name	Sequence
Primers for SARS-CoV-2	F3-SARS-CoV-2	GCCAAAAGGCTTCTACGCA
	B3-SARS-CoV-2	TTGCTCTCAAGCTGGTTCAA
	FIP-SARS-CoV-2	TCCCCTACTGCTGCCTGGAG-GCAGTCAA-GCCTCTTCTCG
	BIP-SARS-CoV-2	TCTCCTGCTAGAATGGCTGGCA-TCTGTCAA-GCAGCAGCAAAG
	LF-SARS-CoV-2	GAACTGTTGCGACTACGTGA
	LB-SARS-CoV-2	GGCGGTGATGCTGCTCT
Primers for MS2 phage RNA [2]	F3-MS2 phage RNA	CCGACAGCATGAAGTCCG
	B3-MS2 phage RNA	AGCCCGCCCACCTTTC
	FIP-MS2 phage RNA	CTCCTGAGGGAATGTGGGAACC CCGGCGTGCGCGTTAT
	BIP-MS2 phage RNA	GCCAGCGAGCTCTCCTCGGGCA CCCGTGCTCTTTCGA
	LF-MS2 phage RNA	GCTGACCGAGGGACCCC
	LB-MS2 phage RNA	GTTAGCCACTCCGAAGTGCG
crRNA for SARS-CoV-2	crRNA-SARS-CoV-2	UAAUUUCUACUAAGUGUAGAU-UUGAACUGUUGCGACUACGUGAU
ssDNA probe	Cas12a-probe-SARS-CoV-2	6-*FAM*-TTATT-BHQ_1

Table S2. The sequence information of four primer sets for LAMP.

Name	Sequence
SARS-CoV-2-F3-1	GCCAAAAGGCTTCTACGCA
SARS-CoV-2-B3 -1	TTGCTCTCAAGCTGGTTCAA
SARS-CoV-2-FIP-1	TCCCCTACTGCTGCCTGGAG-GCAGTCAAGCCTCTTCTCG
SARS-CoV-2-BIP-1	TCTCCTGCTAGAATGGCTGGCA-TCTGTCAAGCAGCAGCAAAG
SARS-CoV-2-LF-1	GAACTGTTGCGACTACGTGA
SARS-CoV-2-LB-1	GGCGGTGATGCTGCTCT
SARS-CoV-2-F3-2	CCAGAATGGAGAACGCAGTG
SARS-CoV-2-B3-2	CCGTCACCACCACGAATT
SARS-CoV-2-FIP-2	AGCGGTGAACCAAGACGCAG-GGCGCGATCAAAACAACG
SARS-CoV-2-BIP-2	AATTCCCTCGAGGACAAGGCG-AGCTCTTCGGTAGTAGCCAA
SARS-CoV-2-LF-2	TTATTGGGTAAACCTTGGGGC
SARS-CoV-2-LB-2	TCCAATTAACACCAATAGCAGTCC
SARS-CoV-2-F3-3	TGGACCCCAAAATCAGCG
SARS-CoV-2-B3-3	GCCTTGTCCTCGAGGGAAT
SARS-CoV-2-FIP-3	CCACTGCGTTCTCCATTCTGGT-AAATGCACCCCGCATTACG
SARS-CoV-2-BIP-3	CGCGATCAAAACAACGTCGGCCC-TTGCCATGTTGAGTGAGA
SARS-CoV-2-LF-3	TGAATCTGAGGGTCCACCAAA
SARS-CoV-2-LB-3	GGTTTACCCAATAATACTGCGTCTT
SARS-CoV-2-F3-4	AGATCACATTGGCACCCG
SARS-CoV-2-B3-4	CCATTGCCAGCCATTCTAGC
SARS-CoV-2-FIP-4	TGCTCCCTTCTGCGTAGAAGCCAATGCTGCAATCGTGCTAC
SARS-CoV-2-BIP-4	GGCGGCAGTCAAGCCTCTTCCCTACTGCTGCCTGGAGTT
SARS-CoV-2-LF-4	GCAATGTTGTTCCTTGAGGAAGTT
SARS-CoV-2-LB-4	GTTCCTCATCACGTAGTCGCAACA

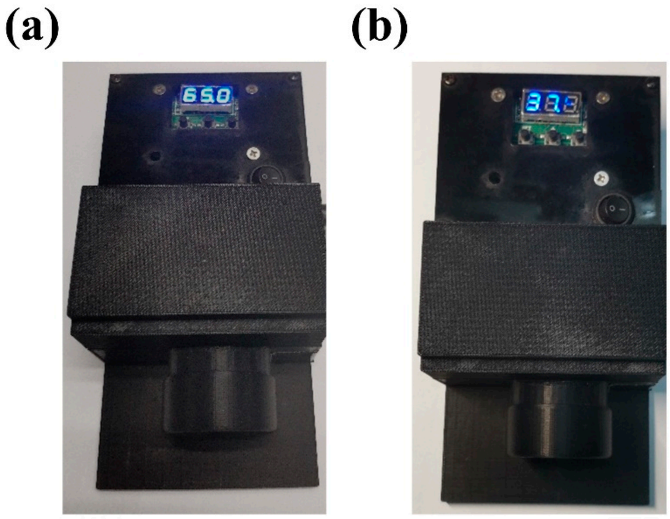

Figure S1. The portable device for LAMP reaction (a), and visualized fluorescence observation (b).

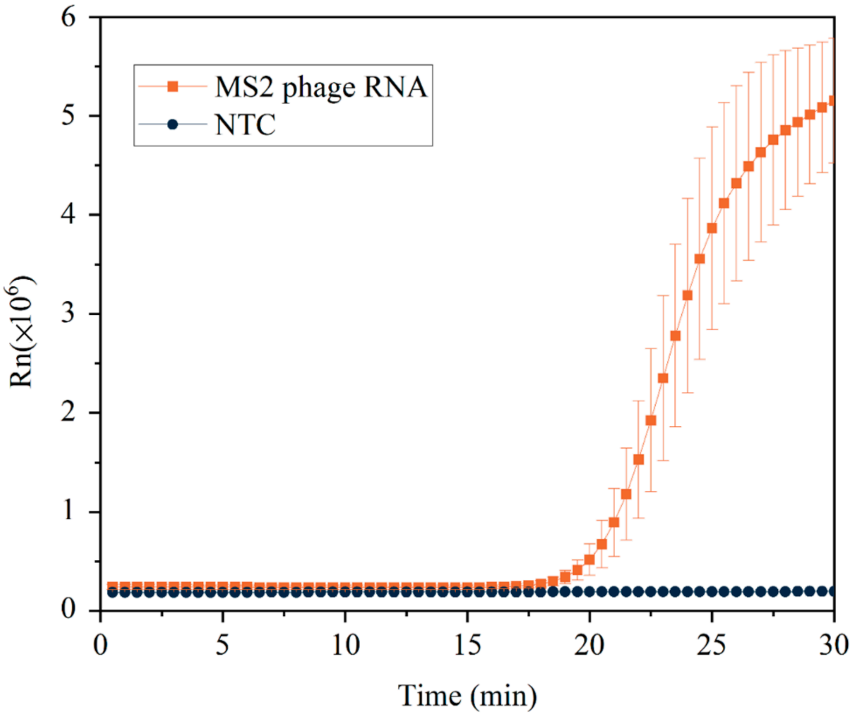

Figure S2. The real-time amplification curves of LAMP for MS2 phage RNA (containing 1000 copies/reaction).

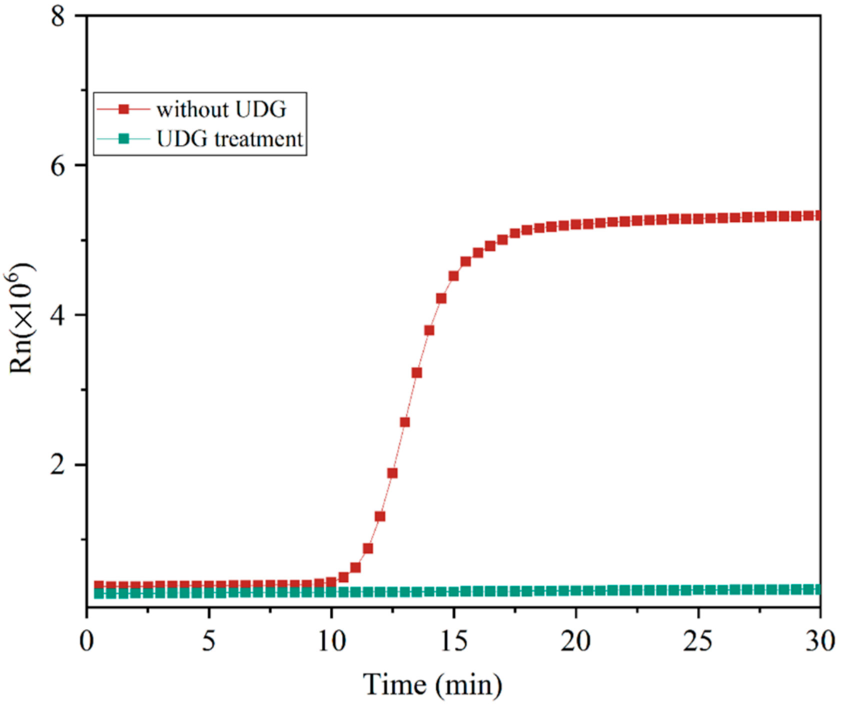

Figure S3. The real-time fluorescence amplification curves of LAMP reaction after UDG treatment and without UDG treatment.

References

[1] R. Wang, X. Xiao, Y. Chen, J. Wu, W. Qian, L. Wang, Y. Liu, F. Ji, J. Wu, A loop-mediated, isothermal amplification-based method for visual detection of Vibrio parahaemolyticus within only 1 h, from shrimp sampling to results, Anal. Methods. **2017**, *9*, 1695-1701. https://doi.org/10.1039/C7AY00165G.

[2] I.P. Oscorbin, G.Y. Shevelev, K.A. Pronyaeva, A.A. Stepanov, D.V. Shamovskaya, O.V. Mishukova, D.V. Pyshnyi, M.L. Filipenko, Detection of SARS-CoV-2 RNA by a Multiplex Reverse-Transcription Loop-Mediated Isothermal Amplification Coupled with Melting Curves Analysis, Int. J. Mol. Sci. **2021**, *22*, 5743. https://doi.org/10.3390/ijms22115743.
